# Synchronization of women’s menstruation with the Moon has decreased but remains detectable when gravitational pull is strong

**DOI:** 10.1126/sciadv.adw4096

**Published:** 2025-09-24

**Authors:** Charlotte Helfrich-Förster, Esther D. Domenie, Oliver Mitesser, Thomas Hovestadt, Alberto Ferlin, Thomas A. Wehr, Rodolfo Costa, Sara Montagnese

**Affiliations:** ^1^ Neurobiology and Genetics, Biocenter, Julius-Maximilians University of Würzburg, Würzburg, Germany.; ^2^ Department of Medicine, University of Padova, Padova, Italy.; ^3^ Theoretical Evolutionary Ecology Group, Biocenter, Department of Animal Ecology and Tropical Biology, Julius-Maximilians University of Würzburg, Würzburg, Germany.; ^4^ Field Station ‘Fabrikschleichach,’ Biocenter, Julius-Maximilians University of Würzburg, Würzburg, Germany.; ^5^ Intramural Research Program, National Institute of Mental Health, Bethesda, MD, USA.; ^6^ Institute of Neuroscience, National Research Council, Padova, Italy.; ^7^ Department of Biomedical Sciences, University of Padova, Padova, Italy.; ^8^ Chronobiology Section, Faculty of Health and Medical Sciences, University of Surrey, Guildford, UK.

## Abstract

To increase reproductive success, many species synchronize reproductive behavior with a particular phase of the lunar cycle. The human menstrual cycle has also a period close to that of the lunar cycle, and recent studies suggest a temporary synchrony between menstrual and lunar cycles. Nevertheless, lunar influence on human reproductive behavior remains controversial. Here, we analyzed long-term menstrual records of individual women from the past 24 years and compared them with records from the past century. We show that women’s menstrual cycles recorded before the introduction of light-emitting diodes in 2010 and the extensive use of smart phones significantly synchronized with the Moon, while those after 2010 coupled to the Moon mostly in January. We hypothesize that the high gravimetric forces between the Moon, Sun, and Earth every January are sufficient for this coupling, while the increasing exposure to artificial light at night impinges on synchrony at other times.

## Teaser

In modern times, menstrual cycles run in synchrony with the Moon only during Perihelion and Minor Lunar Standstills.

## INTRODUCTION

In many aquatic species (*1*–*6*) and several terrestrial species (*7*–*13*), reproductive behavior occurs at a particular phase of the lunar cycle, often the full or new Moon. This arrangement increases reproductive success by synchronizing the reproductive behavior of the individual members of a species. In this context, the human menstrual cycle has a period close to that of the 29.5-day period of the lunar cycle, and several older studies have found a relationship between the cycles (*14*–*16*). Women whose cycles approached the period of the Moon have been reported to have the highest likelihood to become pregnant (*17*–*19*), and three studies of US American women selected for a cycle length of 29.5 ± 1 days demonstrated a significant correlation between menses onset and the lunar cycle (*14*–*16*). The first study was performed during the fall of 1977 on 312 female students of the University of Pennsylvania, 68 of whom had a mean cycle length of 29.5 ± 1 days (*14*). The second study was performed during the fall of 1979 on 305 Brooklyn College students, 97 of whom fulfilled the selection criteria (*16*). The third study was conducted in fall of 1976 and spring of 1983 on 127 and 94 University of Pennsylvania students with 40 and 24 students, respectively, fulfilling the selection criteria (*15*). All of the 229 selected students tended to menstruate at full Moon, suggesting that their ovulation had occurred at new Moon (*15*). Another study from 1986 analyzed cycles of 826 female Chinese participants, aged 16 to 25 years, over four lunar months in different seasons and found that 28.3% of menstruations occurred around the new Moon, while, at other times during the lunar month, the proportion of menstruations occurring ranged between 8.5 and 12.6% (*20*). In this case, no selection on a cycle length of 29.5 day was performed, and the study suggests that ovulation in the Chinese women occurred predominantly at full Moon.

A recent longitudinal study of 22 women who continuously recorded the menses onsets for up to 32 years showed that the cycle length varies greatly with age and becomes shorter as women get older (*21*). In line with the earlier studies, menstruation was, at times, linked to either the full or the new Moon when the cycle length was close to the 29.5-day lunar cycle. Although this coupling was only intermittent and lasted only a few months to a few years, circular plots of all 4112 menstruations of the 22 women showed that the onset of menstruation has a significant bimodal distribution with maxima shortly before the full and new Moon. This result was recently confirmed by a large epidemiological study analyzing 26,912 cycles from 2303 European women and 4786 cycles from 721 North American women (*22*). The study found that menstruation occurred most frequently around the full Moon in the North American women, while it occurred between half and full Moon in the European women. The authors concluded that the lunar cycle is a weak but significant zeitgeber for human reproductive cycles. In addition, they proposed that the human reproductive cycle is a “true” endogenous circalunar oscillator (*22*). Like the older studies mentioned above, the two new studies analyzed menstrual data collected mainly in the past century (predominantly between 1950 and 2000, with very little data up to 2020). In contrast, three other studies examined the relationship between the onset of menstruation and the lunar cycle from data collected in this century using cell phone applications, and they were unable to detect a significant correlation between the menstrual and the lunar cycle (*23*–*25*). These studies feed the general skepticism of the scientific community about lunar influences on human biology (*26*).

To address the discrepancies among the different studies, we extended our previous longitudinal study (*21*) to 176 more recently collected long-term records of menses that were created during the present and past century. As in our previous study, we tested not only their synchronization to full and new Moon but also their synchronization to two other gravitational cycles of the Moon. All other previous studies investigated only the relationship between menstrual cycles and the synodic lunar cycle (full and new Moon).

The Moon exhibits three different cycles that affect moonlight intensity and gravity on Earth (fig. S1) (*21*). The full Moon/new Moon luminance cycle (the synodic month) repeats on average every 29.53 days as the Moon moves through its two points of alignment (syzygies) with the Earth and Sun. The perigee and apogee cycle (the anomalistic month) lasts on average 27.55 days and recurs as the Moon moves back and forth between the nearest (perigee) and farthest (apogee) extremes of its elliptical orbit. The lunar standstill cycle (the tropical month) lasts 27.32 days and arises from the declination of the Moon’s orbit relative to the plane of the Earth’s equator, which causes the Moon to move back and forth between its northernmost and southernmost positions in the sky.

The synodic month clearly affects moonlight intensity and gravity (visible in the tides; fig. S1B), while the anomalistic and tropical months mainly affect gravity and to a lesser degree light intensity (fig. S1, C and D) (*27*, *28*). Furthermore, the lunar cycles interact with each other. For example, every year, Moon-Sun-Earth syzygies coincide with lunar standstills around the times of winter and summer solstices. Their coming into phase with each other at those times is mutually reinforcing and increases the impact of the Moon on the amplitude of the tides at the Earth’s surface in summer and winter.

Furthermore, the distance between the Earth and the Sun affects gravitation on Earth. Each year, the Earth reaches its closest distance to the Sun (Perihelion) between 2 and 6 January; it reaches its farthest distance between 2 and 6 July (Aphelion). During Perihelion, the gravitational forces of the Sun on Earth add to those of the Moon, leading once more to higher tides (*29*). The latter is reinforced by the coincidence of Moon-Sun-Earth syzygies with lunar standstills at the winter solstices in the Northern Hemisphere.

In addition to these seasonal gravitational cycles, there are two gravitational cycles lasting ~18 years, which result from the interactions between the Moon’s orbit around the Earth and the Earth’s orbit around the Sun and are described in further detail in the pertinent results sections.

In the present study, we found evidence that the menstrual cycle may meet the criteria of a circalunar clock that entrains (actively synchronizes) to all three lunar cycles: the synodic, anomalistic, and tropical months. As for all endogenous clocks, the entrainment depended on the strength of the zeitgeber. Before 2010, there was a clear entrainment to the full or new Moon at the individual and population level (when all menstrual periods were pooled), whereas, after 2010, when artificial light at night increased markedly, this entrainment was no longer detectable in the pooled data. Nevertheless, it persisted when the gravitational forces of the Moon and Sun were mutually reinforcing: (i) during solstices coinciding with Perihelion in December/January, (ii) during the times when the Minor Lunar Standstill coincided with Saros cycle #137, and (iii) to lesser degree during Major Lunar Standstill. In addition, a weak but significant synchronization of the onset of menstruation with the gravitational cycles of the Moon (the anomalistic and tropical months) was found not only at the individual but also at the population level during winter and summer solstices.

We conclude that humans are affected by the luminescence cycles and also the gravimetric cycles of the Moon, with the menstrual cycles aligning with the synodic month. Furthermore, we hypothesize that, in ancient times, human reproductive behavior was synchronous with the Moon, but that our modern lifestyle, particularly our increasing exposure to artificial light at night, has altered this relationship.

## RESULTS

**Fig. 1.**
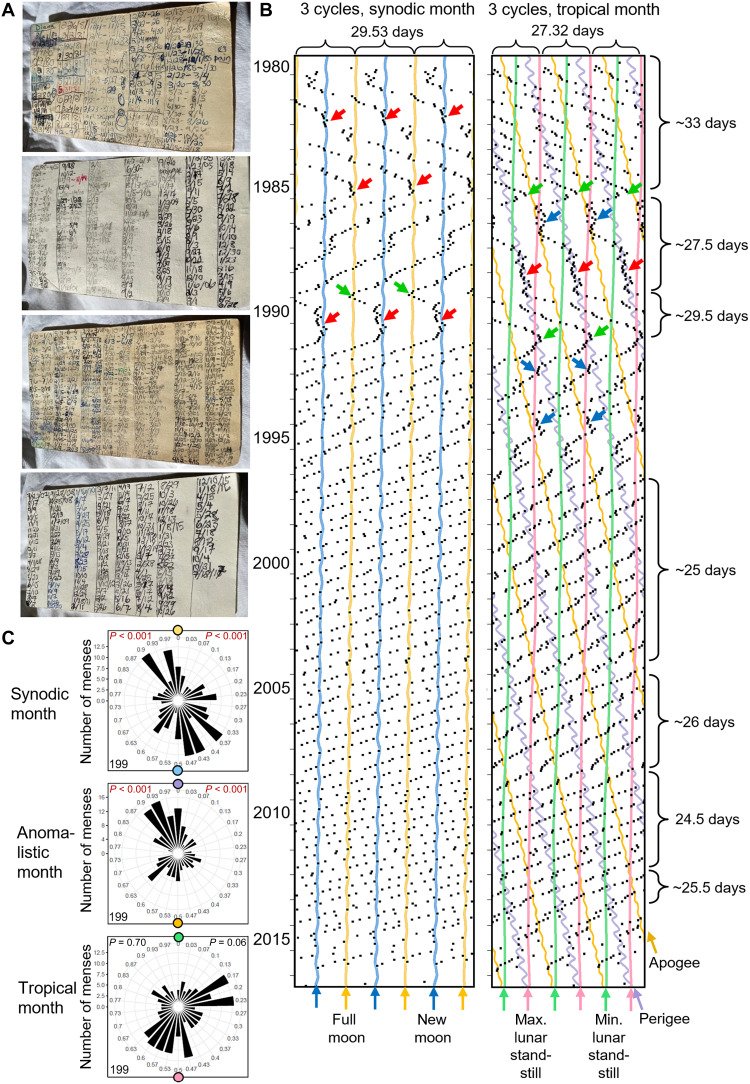
Thirty-seven-year record showing the long-term characteristics of menstrual cycles in relation to lunar cycles. (**A**) Original menses record (from menarche at 13 to menopause at 50 years of age) from a North American woman. (**B**) Mensograms plotted with the approximate period (29.53 days) of the synodic month (left) and the tropical month (27.32 days) (right). The plots are repeated three times to facilitate visual inspection of the menstrual cycle courses. Consistent with Helfrich-Förster *et al.* (*21*), onsets of menses are shown as black dots, while times of full Moons and new Moons are indicated as yellow and blue undulating lines (left), and those of maximum and minimum lunar standstills as pink and green lines (right), respectively. Perigees (lilac strongly undulating line) and apogees (orange line) of the anomalistic month (27.55) are also indicated in the right mensogram. The menstrual cycle coupled temporarily to full Moons, new Moons, and perigees (red arrows) as well as an association with minimum lunar standstill (blue arrows), which was not significant in the polar plots (**C**). Furthermore, it showed sudden changes in period that sometimes coincides with full Moon, apogee, or perigee (green arrows). From the beginning to the end of recording, the period shortened from 33 to 24.5 days. From a period of ~25 days onward, it was no longer synchronized with any lunar cycle. (C) Circular plots of the distribution of menses phases throughout the synodic, anomalistic, and tropical cycles for the first 15 years of recording. Rayleigh tests were performed to check for the presence of a uni- or bimodal distribution with the phases synchronized to the full/new Moon, perigee/apogee, or minimal/maximal lunar standstill (left *P* values) and for deviations from a uniform frequency distribution of any phase (right *P* values). Numbers of recorded menses are shown in the lower left corner of each graph.

In total, we collected 176 menstrual records over periods of 2 to 37 years (mean, 6 years) from women who were not using oral contraceptives. Unlike in our previous study, most women started recording their menstruation at an age younger than 30 years (mean, 25.9 ± 6.4 years); eight women even started recording at menarche. Sixty records were from the past century, 111 were from this century, and 5 spanned both centuries. As in our previous study, most of the records were from women living in Europe (mainly in Italy or Germany), but we also obtained 9 records from other European countries, 10 records from Israel, and 5 records from women living in North America (Table 1). Records from the past century were noted on paper, either as crosses in calendars or as a list of menses onsets (and sometimes also menses offsets) (see Fig. 1A). The great majority of menses from this century were recorded with cell phone apps, and either sent as screen shots or as Excel or Word files.

**Table 1.** Origin and characteristics of menses recordings.

Country	Total number	Number past century	Number this century	Number spanning both centuries	Mean recording duration (± SD)	Mean age recording begin (± SD)	Mean cycle period (± SD)
Italy	86	31	54	1	4.6 ± 3.6	27.4 ± 6.0	28.4 ± 2.9
Germany	66	29	35	2	6.1 ± 6.3	24.1 ± 6.5	29.5 ± 2.4
Rest of Europe	9	–	9	–	6.2 ± 2.7	26.7 ± 6.0	30.0 ± 3.9
Israel	10	–	10	–	5.6 ± 2.5	26.8 ± 6.8	27.9 ± 2.2
North America	5	–	3	2	19.1 ± 12.4	25.8 ± 9.6	29.8 ± 1.0

To determine whether menses onsets were synchronous with the lunar cycle, we analyzed the relationship of menses onsets to the full Moon and the new Moon, to minimum and maximum lunar standstills, and to lunar perigees and apogees for each individual. Menstrual data were displayed as raster plots (mensograms), together with lines depicting the courses of the full and the new Moon or of minimum and maximum lunar standstills and perigees and apogees. The menstrual data also were subjected to time series analysis (*30*).

### Evidence for the menstrual cycle being a circalunar clock with a limited range of entrainment

**Fig. 2.**
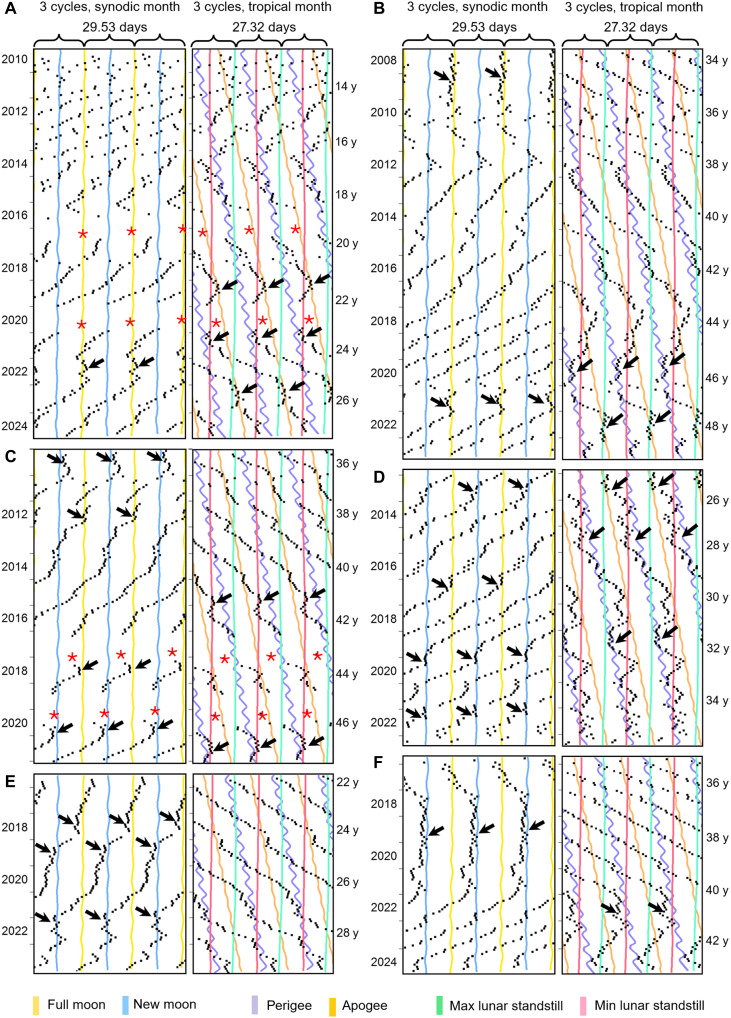
Menstrual periods show a high degree of individual variability and determine the probability of a coupling to the lunar cycles. (**A**) Mensogram of a woman who recorded her menses from menarche onward and started with an unusually short period. Over the years, period slowly lengthened and coupled to apogee at the age of 21 and to minimum lunar standstill (and perigee) at the age of 23 (arrows). At the age of 25, her menses was loosely entrained to full Moon for almost an entire year, and, afterward, it appeared to couple again to apogee. (**B**) Mensogram of a woman, who started recording her menses at an age of 34 and was entrained to full Moon for the first 2 years of recording (arrow). At the age of 47, she was again entrained to full Moon for about 1 year (arrow). Before, she was coupled for 1 year to perigee and later to maximum lunar standstill (arrows). (**C**) Mensogram of a woman, who started recording her menses at an age of 36 and coupled several times briefly to new or full Moon as well as to minimal lunar standstill during the next 10 years. Red asterisks indicate births. (**D**) Mensogram of a woman, who maintained a consistent period of about 27.1 days for about 10 years. Her menses coupled several times briefly to new or full Moon as well as to minimal lunar standstill and perigee. (**E**) Mensogram showing brief couplings to full and new Moon. (**F**) Mensogram showing a long coupling to new Moon followed by a loose coupling to perigee/minimal lunar standstill. At the end of recording, the menses occurred again close to full Moon. Labeling as in Fig. 1. y, years.

Figure 1 shows the longest record, which comes from a woman in North America. She recorded her menstruation for 37 years from menarche to menopause (a total of 497 menstruations), and her mensogram is typical in several respects. First, she started with a long period of her menstrual cycle (~33 days), which then continuously shortened (24.5 days at an age of 43 years), until at the end, before it became irregular, it lengthened again (25.5 days). Second, her cycle showed irregularities with sudden jumps and changes in period (green arrows). Third, in her younger years, her cycle was intermittingly coupled to the new and full Moon of the synodic month and to perigee of the anomalistic month (red arrows in Fig. 1B and pointed distribution in Fig. 1C). Fourth, from 1995 onward, when she had reached an age of 30 years and the period of her cycle was ~25 days long, the coupling to the lunar cycles disappeared. The latter observation fits with our previous study (*21*), which showed that the coupling to lunar cycles became weaker with age, when the period of the menstrual cycle shortened beyond a certain limit. In the present study, we observed the same phenomenon and also obtained evidence that the coupling to the lunar cycles did not directly depend on age, but rather on the period of the menstrual cycle. Even women over 35 years of age were able to couple to the synodic month if their periods were longer than 26 days (Figs. 2, B, C, and F, and 3A). Nevertheless, the likelihood of coupling to the synodic month was higher in young women and appeared particularly high in the first 5 years after menarche. The period of the menstrual cycles of the eight women who recorded their menses onsets from menarche onward was on average 32 days (the woman shown in Fig. 2A exhibits an exceptionally short period of her menstrual cycle). Although the menstrual cycle was still irregular at a young age (see Fig. 1B from 1980 to 1985 and Fig. 2A from 2010 to 2015), it coupled intermittently to the synodic month in six of the eight women.

**Fig. 3.**
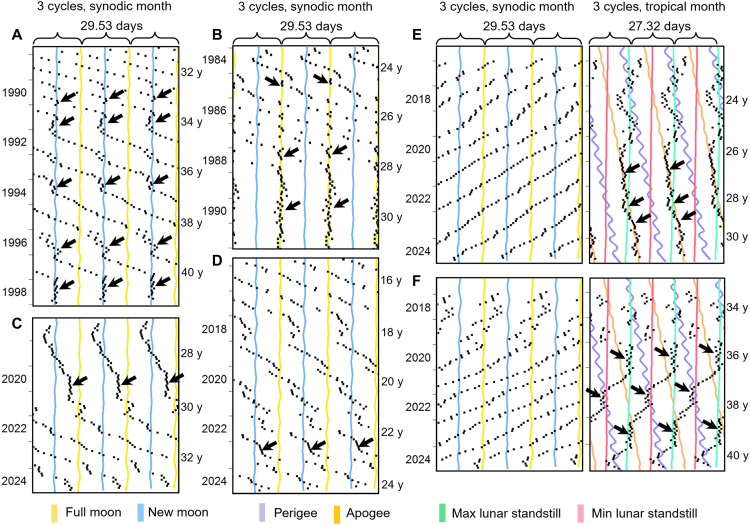
Mensograms showing relative coordination and stable entrainment. (**A** to **D**) Menstrual cycles with long period that showed either relative coordination to new Moon (A), relative coordination and stable entrainment to full Moon (B), intermittent entrainment with a very early phase (C), or something between relative coordination to new Moon and free-run (D). (**E** and **F**) Menstrual cycles with short period that did not entrain to the synodic month but showed either stable entrainment to the anomalistic month (E) or relative coordination to maximum and minimum lunar standstill (F). Labeling as in Fig. 1. y, years.

At very long periods (>33 days), the menstrual cycle sometimes showed “relative coordination” to the synodic month (Fig. 3, A and B). Relative coordination is a chronobiological term, which means that a rhythm alternates between a free-run and synchronization (entrainment). This phenomenon is typical for biological clocks (oscillators) that are at the limit of their entrainment range [e.g., (*31*)]. Consistent with this, we also found relative coordination when the lunar clock had a short period (<27 days) and was at its limit of entrainment to the synodic month (Fig. 2D). The same happened at the limits of entrainment of the anomalistic and tropical months. An illustrative example for the tropical month with rather long phases of entrainment is shown in Fig. 3F. Here, the menstrual cycle entrained intermittingly to minimum and maximum lunar standstills.

**Fig. 4.**
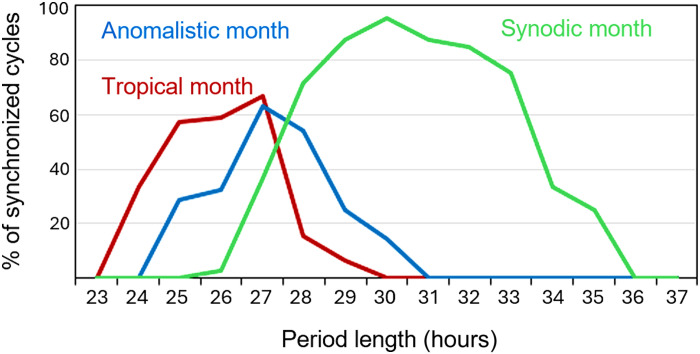
Limits of entrainment to the synodic, anomalistic, and tropical months. For each woman and for each episode with a consistent free-running period, it was determined whether or not menstruation occurred synchronously with one of the three lunar months in at least four consecutive cycles (yes or no). This synchrony could occur before or after episodes with a consistent free-running period. The number of positive events (“yes”) was then expressed as a percentage of all episodes examined and plotted against the free-running period.

We defined the limits of entrainment of the menstrual cycle for all three types of lunar months by determining the percentages of cycles that intermittingly coupled (for at least 4 consecutive months) to the synodic, anomalistic, and tropical months at different free-running periods (Fig. 4). We found that the percentage of entrained cycles was clearly dependent on the free-running period that they had before or after the entrained interval. For the tropical month the range of entrainment reached from 23 to 30 days, for the anomalistic month from 24 to 31 days, and for the synodic month from 26 to 36 days (Fig. 4). This strengthens the conclusion of Ecochard *et al.* (*22*) that the menstrual cycle is governed by a true endogenous circalunar oscillator. The fact that this circalunar oscillator has its largest range of entrainment for the synodic month (Fig. 4) suggests that the synodic month is its strongest zeitgeber of the three lunar months.

**Fig. 5.**
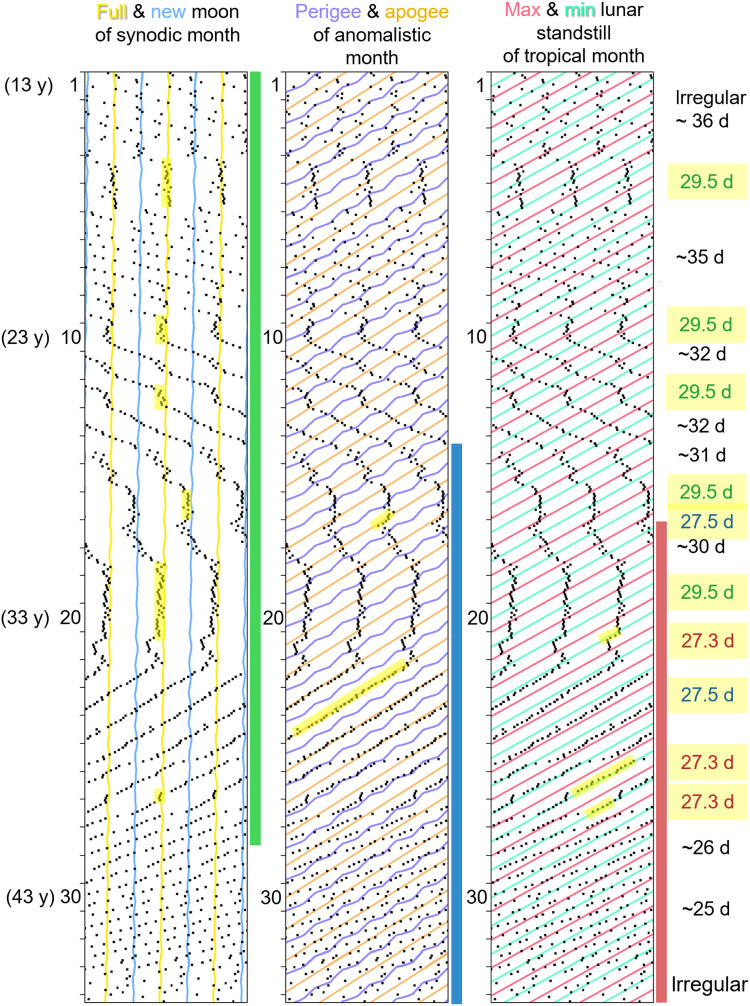
Typical model mensogram showing three decades of menstruation for a fictional woman without births. All three mensograms are identical, plotted with the period of the synodic month [29.5 days (d)] and overlaid with the three different lunar cycles (see explanation on top). The maximal ranges of entrainment are taken from Fig. 4 and shown as colored bars on the right side of the mensograms. Times of entrainment are marked in yellow in the mensograms and calculated periods are shown on the right. After menarche [at 13 years (y) of age] the menstrual cycle is usually irregular and free-runs with rather long period, which shortens continuously with age until it becomes irregular and sometimes longer again before menopause (at 46 years of age). Within the entrainment range for the synodic month, menstruation is intermittently synchronous with the full moon five times and with the new moon once, while it is synchronous with the apogee twice within the entrainment range of the anomalistic month and three times with the minimum lunar standstill within that of the tropical month.

Our hypothesis of the existence of a circalunar clock that changes period over the lifespan and entrains intermittently to all three lunar cycles is illustrated in a model mensogram from menarche to menopause built from four different women (Fig. 5). Initially, the cycle is irregular with a long free-running period, but it can temporarily entrain to the synodic month due to its large range of entrainment. With time, the period of the menstrual cycle shortens, to become very short and irregular before menopause. Periods within a range of 26 to 30 days can entrain to each of the three lunar months (Fig. 5).

On average, the free-running period of all 176 women and at all times was ~29 days, which was similar to our previous study. Nevertheless, we observed some regional differences (Table 1). Italian and Israeli women had on average a period that was ~1 day shorter than that of women living in the other countries (Table 1). One putative reason for this shorter period might be age, as the Italian women were slightly older than those living in the other countries (Table 1). However, this was not true for the Israeli women, who started recording at the same age as most of the other women. Other possible reasons might be latitudinal differences or light pollution, which appeared higher in North Italy and Israel (*32*) and is known to shorten period between menses (*33*–*35*) (see the next section and Discussion).

To investigate whether the estimated date of conception, childbirth, and the first menses after childbirth preferentially occurred at certain Moon phases, we analyzed these events separately. We observed strongly peaked distributions for all three events either around full Moon and/or new Moon (fig. S2), but, most likely in relation to the small number of cases (36 births), this result was not significant.

### Entrainment to the synodic month decreased during recent years

When comparing the mensograms recorded during the past 10 to 15 years with those recorded earlier (e.g., Fig. 3, A and B), we noticed that entrainment to the synodic month was still present (Fig. 2) but that the phases of menses onsets relative to the full or new Moon were more variable than earlier and did not always occur about 1 day before the full or new Moon (e.g., Fig. 3C). In contrast, we more often observed entrainment to apogees or minimum and maximum lunar standstills (Fig. 3, E and F).

**Fig. 6.**
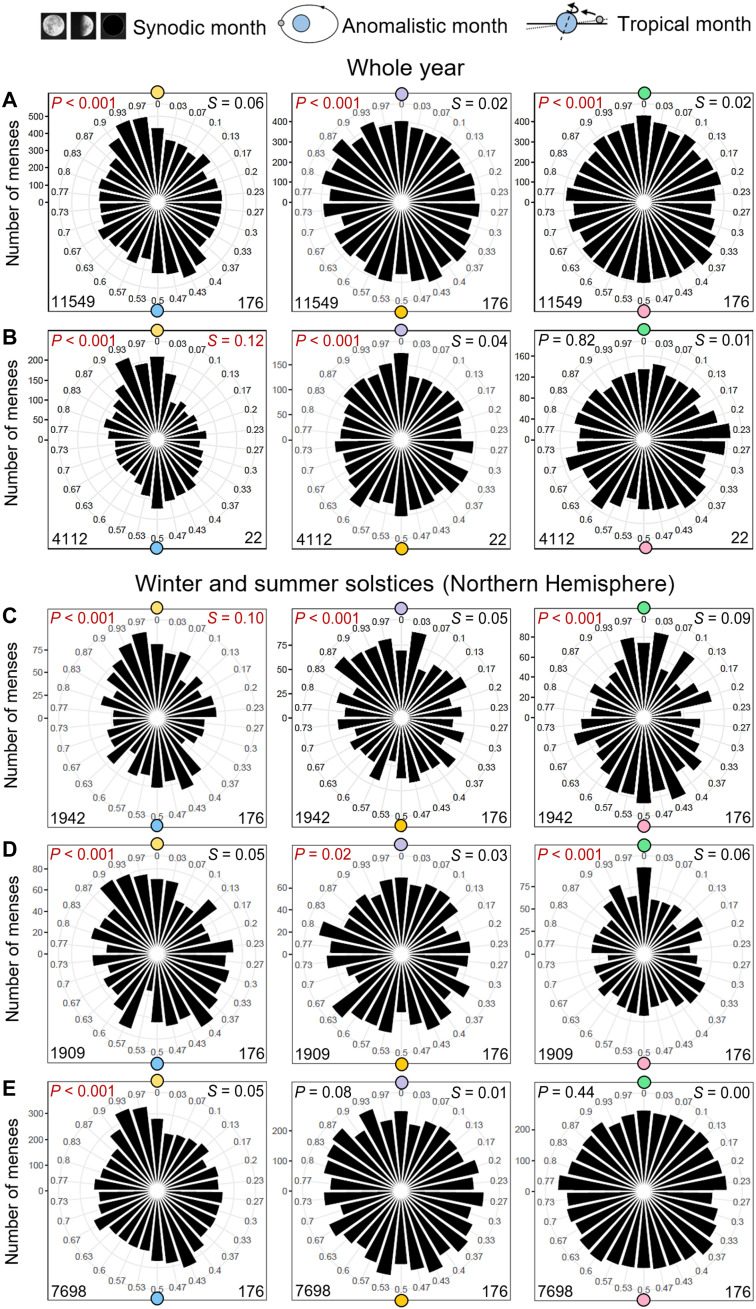
Menstrual cycle phases are aligned with the phases of full/new Moon, perigee/apogee, and minimal/maximal lunar standstill, and this alignment depends on the season. Circular phase plots show the phase relation between menstrual cycles and the synodic, anomalistic, and tropical months. These plots combine all 11,565 menses of the recorded 176 women of the present study (**A**), and, for comparison, the circular phase plots derived from our old study (*21*) with 4112 menses of 22 women are shown (**B**). In contrast to the old study, which showed only significant alignments with the time of the full or the new Moon and with the time of perigee or apogee but not with the time of lunar standstills, the new study found a significant alignment with all three lunar cycles (*P* values of Rayleigh tests given in the top left corners). Nevertheless, the circular plot for the synodic month of the new study was less peaked than that of the old study (S values that indicate the length of the directional vector are given in the top right corners; the lower the number, the less peaked is the distribution; we regard *S* values of ≥0.10 as highly peaked distributions). (**C** to **E**) Phase relation between menstrual cycles and the synodic, anomalistic, and tropical months during the winter solstice (C), the summer solstice (D), and the rest of the year (E). The phase distribution was always more peaked during the winter solstice, and, in the case of the anomalistic and tropical months, a peaked distribution was only present during the winter and summer solstices.

To see, whether coupling to the lunar cycles is still significant at the population level, we calculated polar histograms. These included the menses of all 176 women regardless of the century of recording and regardless of whether a synchrony with the different lunar cycles is visible in the mensograms. These polar plots confirmed that the phase distributions of the menses still had a preferential direction and were not uniformly distributed. Circular statistics showed a significant bimodal distribution to full and new Moon, to perigee and apogee, as well as to minimum and maximum lunar standstill (Fig. 6A). This bimodal distribution was most pronounced for the synodic month.

When compared to our previous results with the 22 women (Fig. 6B), we saw many similarities but also some interesting differences. The bimodal distribution to full and new Moon was less peaked in the new data. Furthermore, it was slightly phase-shifted with the maxima occurring 2 days instead of 1 day before the full and new Moon, respectively. The distribution to apogee and perigee of the anomalistic month also appeared slightly less peaked than in the old data. However, the distribution to minimum and maximum lunar standstill was more peaked and reached statistical significance, whereas this was not the case in the old data (Fig. 6, A and B).

Together, those observations suggest that entrainment to the synodic month was less strong in the new data, while entrainment to the tropical, purely gravimetric lunar cycle was slightly enhanced. A putative reason for these differences is the increasing light pollution, which weakens the impact of luminescence cycles of the Moon but leaves the impact of gravimetric cycles unchanged. If this is true, then synchrony with the lunar cycles should be greater during the winter and summer solstices than during the rest of the year, because the gravimetric cycles are most pronounced at these times. This was the case (Fig. 6, C to E): At the population level, significant synchronization of menstrual cycles with the anomalistic and tropical months persisted only during the winter and summer solstices and disappeared during the rest of the year, while menses distribution in relation to the synodic month was more peaked during the winter solstice than at other times of the year. Also for the anomalistic and tropical months, menses distribution was more peaked during the winter solstice than during the summer solstice, which could be caused by the temporal coincidence with the perihelion.

In addition, the differences in the entrainment to the synodic month should be particularly pronounced, when the new data are separately analyzed for times with lower and higher light pollution. Measurements of global radiated power carried out by satellites between 1992 and 2017 have shown that light emission from Earth increased steadily, but that there was a notable increase from 2010 on, when light-emitting diodes came onto the market and replaced conventional light bulbs (*32*, *36*). At the same time, the use of mobile phones, smartphones, and other screens increased markedly. These devices were no longer used just for making phone calls, but for practically everything that could be done online, from social interactions to banking and geographical navigation.

**Fig. 7.**
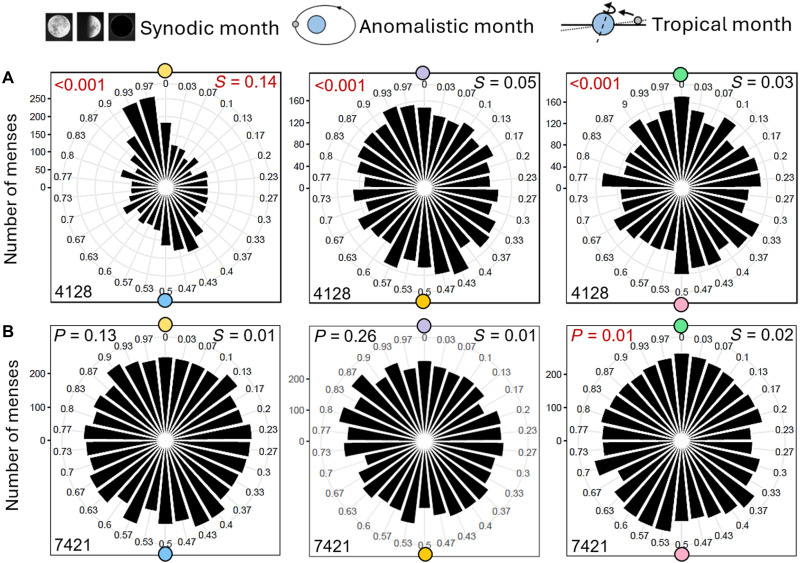
Light pollution is a likely cause for the loss of a significant alignment of the menstrual cycles with full and new Moon in the data recorded after 2010. (**A** and **B**) Polar phase plots of the menses recorded before 2010 (A) and after 2010 (B) show that the alignment to full and new Moon disappears after 2010, while the alignment to minimal/maximal lunar standstill remains similar.

Therefore, we decided to analyze the new data collected before and after 2010 separately. This analysis showed that synchronization to the synodic month was completely lost on the population level after 2010, while those to the purely gravimetric tropical month remained similar (Fig. 7, A and B).

Moreover, a separate analysis of the data from the German and Italian women revealed that the distribution to full and new Moon was more peaked for the menses of the German than the Italian women (fig. S3). Not only was the distribution of menstruation less peaked in Italian women, but it was also more bimodal with equal distribution toward 2 days before full and new Moon, whereas the distribution of menstruation in German women was clearly skewed toward 1.5 days prior the full Moon (fig. S3). These results fit well with our hypothesis that light pollution contributes to the decline in menstrual synchronization with the synodic month, as light pollution is higher in Northern Italy (fig. S3), where most of the data comes from, than in Germany (*37*). Nevertheless, this remains hypothetical as data on actual light exposure of women included in this study are not available.

### Long-period gravimetric lunar cycles reinforce entrainment to the synodic month

Every 18 years and 10.33 days (18.03 years), the Moon passes exactly the same node at which its orbital plane intersects with the ecliptic Earth-Sun plane and additionally a full or new Moon occurs (=Saros series) (*21*). If total eclipses and a close perigee distance characterize a certain Saros series, then the gravitational forces of the Moon on Earth are maximal. In our previous study, we identified a certain Saros series (Saros #137) during which the Moon was exceptionally close to Earth and during which most of the women were in synchrony with full or new Moon (*21*). This Saros series occurred in 1961, 1979, 1997, and 2015.

Another circa 18 years long cycle (in this case, 18.61 years) occurs in the declination of the Moon’s orbital plane around the Earth in relation to the ecliptic (Earth’s orbital plane around the Sun), which varies between 18.134° (north or south) and 28.725° (north or south) due to lunar precession. These extremes are reached every 9.3 years and called the Minor and Major Lunar Standstills (or lunistices), respectively (fig. S4). Depending on location and the form of the tides, Minor or Major Lunar Standstills cause high-amplitude tides and tidal flooding (*38*, *39*). In locations at which the tides occur twice every day (in a 12.4-hour rhythm) Minor Lunar Standstills leads to high-amplitude tides, while in locations at which the tides occur only daily (every 24.8 hours) Major Lunar Standstills cause flooding (*29*). The last four Minor Lunar Standstills occurred in 1959, 1978, 1997, and 2015, which is very close to Saros series #137 (see above). Major Lunar Standstills reached its peaks in 1969, 1987, 2006, and 2025.

To see whether Minor and Major Lunar Standstills affect the synchronization of the menstrual cycle to the synodic month, we calculated polar histograms of menses onsets for 3-year intervals around Minor and Major Lunar Standstills, respectively (for the Major Standstill that occurred in March 2025, we used the years 2023–2024). We chose these intervals because the Moon’s declination changes very little during those periods (www.umass.edu/sunwheel/pages/moonteaching.html). Because the new data of the 176 women contained very few values between 1950 and 1980, we added the data from the 22 women of our old study.

**Fig. 8.**
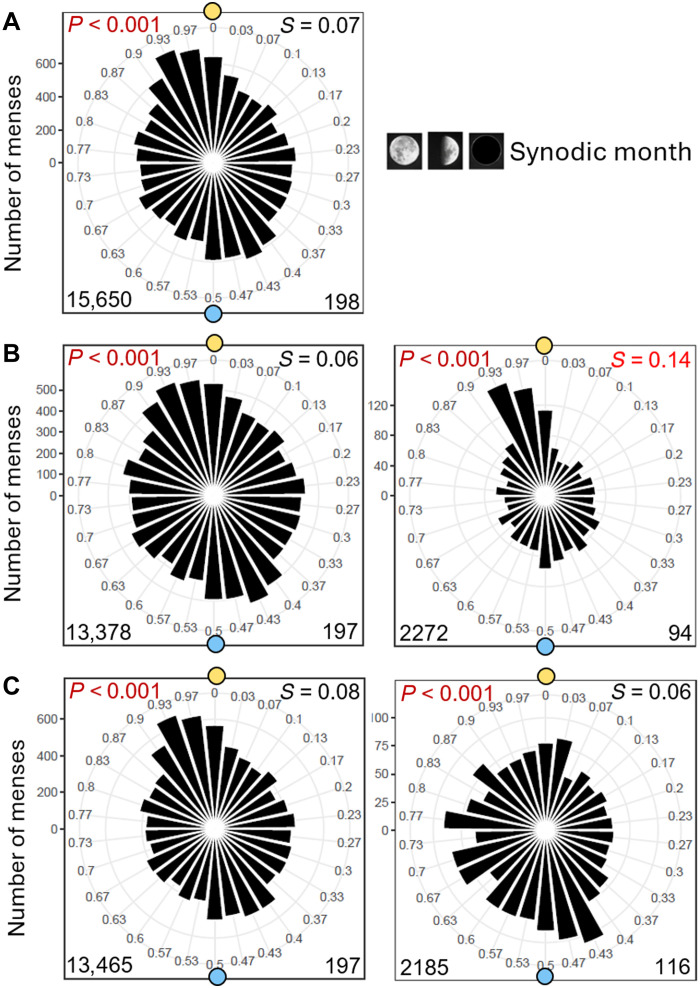
The alignment of menstrual cycles with full Moon is very strong during Minor Lunar Standstill. (**A**) Polar phase plots of all menses onsets (including the old and new study) relative to synodic months for the entire recorded period (1950–2024). (**B**) Menses distribution outside (left) and within 3-year intervals around Minor Lunar Standstill (1979, 1997, and 2015) (right). (**C**) Menses distribution outside (left) and within 3-year intervals around Major Lunar Standstill (1987, 2006, and 2025). For the Major Standstill that occurred in March 2025, we used the years 2023–2024. Labeling as in Fig. 6.

We found that menses had a very peaked distribution and accumulated around full Moon during the 3-year intervals of the Minor Lunar Standstill, while their distribution was less peaked during 3-year intervals of the Major Lunar Standstill, when they accumulated around new Moon (Fig. 8). When performing the same analysis for the anomalistic and tropical months, we detected only minor differences in menses distribution during Minor and Major Lunar Standstill for both cycles (figs. S5 and S6).

### Google Trends reveal increased queries for menstrual cycle–related words during perihelion

**Fig. 9.**
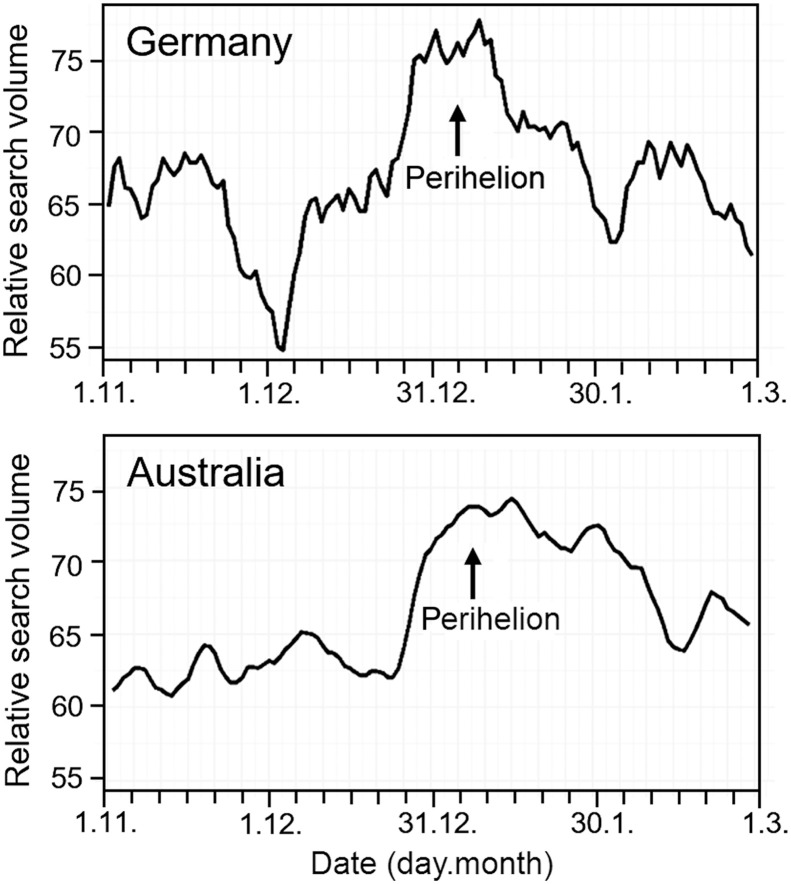
Google Trends reveals increased queries for period pain during perihelion. Average daily RSV for the search term period pain in Germany and Australia from 1 November to 1 March over years 2013–2024. An increase in RSV is visible at the beginning of January, i.e., around perihelion (which is at the beginning of January, with minor variations on the exact date in different years), in both countries.

Trends in Google searches for “period pain” and pertinent translations were analyzed in the United Kingdom, Germany, Sweden, Italy, The Netherlands, and France (for the Northern Hemisphere) and South Africa, Australia, and New Zealand (for the Southern Hemisphere) from 1 November to 1 March for the years 2013–2024 (i.e., 4 months around perihelion) and over the 2 months before/after the spring and autumn equinoxes in 2015 (20 March and 23 September), when the Saros #137 and Minor Lunar Standstill coincided. Relative search volumes (RSVs) of Google queries within a given timeframe and geographical location are accessible through Google Trends (https://trends.google.com/trends/). The RSV is calculated from an anonymized representative random sample of the total search frequency, while absolute values are not accessible. RSV comparisons in different geographical locations are possible, again only in relative terms. When search queries are not very popular or analyzed over small regions, individual random samples used to calculate the RSV might vary considerably. To overcome this issue, RSVs were extracted using the package trendEcon (R-studio, version 4.2.1; https://trendecon.github.io/trendecon/articles/intro.html), which queries Google Trends multiple times with a moving time window, thus allowing extraction of more stable time series with a daily frequency while also preserving the weekly and monthly trends. The rolling average of daily RSVs for 1 November to 1 March for the years 2013–2024 was computed. Comparisons between the countries originally selected documented higher RSVs for Germany in the Northern Hemisphere and Australia in the Southern Hemisphere, which were, therefore, retained as most informative examples. Obvious increases in RSV for the search term period pain were observed around perihelion (i.e., the beginning of January) in both Germany and Australia (Fig. 9).

The same trends were obvious also in the other countries, with the exception of Italy, France, and New Zealand. In all these instances, RSVs were lower in the countries for which trends were not observed (or were less obvious) compared to the remaining countries. RSVs around 2015 equinoxes were not consistent. In summary, the above Google Trends research outputs seem to confirm, in a totally independent fashion compared to the rest of the data presented here, the association between lunar gravimetric effects and menstrual cycles.

## DISCUSSION

We provide evidence that the menstrual cycle fulfills some criteria of a circalunar clock, which can entrain (actively synchronize) to all three types of lunar cycles: the synodic, anomalistic, and tropical months. Internal (endogenous) clocks are defined as self-sustaining biological oscillators that continue to run under constant conditions with a species-specific free-running period. Endogenous clocks are temperature compensated (i.e., their free-running period is independent of the ambient temperature), and they entrain to the environmental cycles and even follow their phase shifts. They are thought to have an adaptive function. Not all these principles are fulfilled by a hypothetical menstrual lunar clock and/or cannot be experimentally verified. For example, it is impossible to record the menstruation cycle over several years under constant conditions. Nevertheless, the fact that menstruation cycles free-run under cyclic environments is a strong argument in favor of their being self-sustaining biological oscillators. An experimental phase shift of the lunar cycles and the subsequent reentrainment of the menstrual cycle appears also impossible to test, and testing for temperature compensation is generally difficult in homeothermic animals.

The most important argument in favor of the existence of a circalunar clock that controls the menstrual cycle is its limited range of entrainment and its responsiveness to light. A limited range of entrainment means that entrainment can only happen when the endogenous period of the menstrual cycle is not too far from the periods of the lunar cycles. Here, we were able to define limits of entrainment to all three types of lunar months. In addition, we show that menstrual cycles at the limits of their entrainment range exhibit relative coordination with the lunar cycles. Relative coordination can only be explained by the interaction of an endogenous clock with its zeitgebers. Further, like the human circadian clock, the circalunar clock is strongly influenced by light and internal factors. Light at night appears to shorten the period of the menstrual cycle (*33*–*35*), while it lengthens that of the circadian sleep-wake cycle (*40*). We and others have shown that the period of the menstrual cycle shortens with age (*21*, *41*, *42*), while Roenneberg and colleagues (*43*) were the first to demonstrate that the circadian chronotype shifts to later phases during puberty and then becomes progressively earlier with increasing age, a phenomenon that has been confirmed by later studies and may relate, to some extent, to age-dependent changes in sex hormones (*44*–*46*). Our findings considerably extend the results of Ecochard *et al.* (*22*), who showed that the menstrual cycle varies dynamically around the average cycle length, suggesting that menstrual cycles may be controlled by a circalunar clock.

We found that the synodic month is the strongest zeitgeber for the menstrual cycle. Nevertheless, even the synodic month acts as a rather weak zeitgeber, because the menstrual cycles continuously entrain to it, at most, for a few years and in most cases for several months, although sometimes repeatedly. After the exposure to artificial light at night increased markedly, the menstrual cycles no longer showed entrainment to the synodic month at the population level. This observation is consistent with the lack of synchrony observed in the mass analyses of menstruation cycles recorded by cell phone apps, which all took place after 2010 (*23*–*25*). Nocturnal exposure to artificial light not only impairs the sensing of moonlight, but also shortens the period of the menstrual cycle (*33*–*35*), which makes entrainment to the synodic month more unlikely.

### The influence of gravimetric forces

Despite the loss of population synchrony to the synodic month in recent times, we see an unaltered significant accumulation of menses onsets around the full and new Moon during the winter months (December and January), which is accompanied by increased searches for menses-related issues on Google Trends around perihelion in several countries. At perihelion, the Earth is closest to the Sun, and, around the winter solstice, the full and new Moons coincide with maximum and minimum lunar standstills, respectively. Relationships between syzygies and standstills are reversed around the summer solstice, while the Earth is at its farthest distance from the Sun (aphelion). Aphelion may explain why we see a less peaked accumulation of menses onsets around the full and new Moon during June and July. The fact that the gravitational influence of the Moon on the Earth is increased in December/January could explain why, in most countries, the search for period pain is greatest at this time. This was similar in the Northern and Southern Hemispheres, although December/January in the Southern Hemisphere are characterized by long summer days. This does not support the hypothesis that short winter nights, when the moon is more visible, are the reason for better synchronization of menstrual cycles with the synodic months. Nevertheless, there may be other seasonal factors (both environmental and social) that contribute to menstrual cycle synchronization in December and January.

In addition to the seasonal influences of the Moon on menses, we found a notably peaked distribution of menses onsets toward the full Moon around the every 18.6 years occurring Minor Lunar Standstills and a less peaked distribution to the new Moon in the years around Major Lunar Standstills. The strongly peaked distribution of menses onsets during Minor Lunar Standstills can be explained by the simultaneous occurrence of the Saros series #137, which further intensified the gravimetric influences of the Moon on Earth.

Together, this indicates that that the lunar gravimetric forces enhance the entrainment of the menstrual cycles to the synodic month. Our observation that gravitation works as a zeitgeber on humans may explain why menstrual cycles, mood cycles (*47*), and cycles in sleep onset and sleep length (*48*) coupled temporarily either close to the full Moon or close to the new Moon, because, at both phases, the Moon’s gravimetric influence on Earth is similar. Effects of gravity might also account for the fact that synchrony of sleep onset and sleep duration with the lunar cycle has been observed in college students living in the light-polluted city of Seattle, where the Moon’s luminance cycle is scarcely perceivable (*48*). Together, these observations raise a possibility that the human organism can respond not only to fast gravitational changes, like those detected by the vestibular system, but also to slow, periodically recurring gravitational changes.

Nevertheless, it seems unlikely that humans can directly perceive the gravitational changes caused by the Moon and the Sun. These changes might be sensed indirectly through changes in geophysical variables. For example, atmospheric pressure cycles are measurable during perigee-syzygy tides (*49*), and there are initial reports that terrestrial plants and animals can sense them (*50*, *51*). In addition, the Moon affects the Earth’s magnetotail, thereby creating oscillating electromagnetic fields on the Earth’s surface (*52*) that can be sensed by animals (*53*, *54*). Perhaps humans can sense all these types of oscillations, as well (*48*, *53*, *55*). Cosmic rays may also oscillate with periods close to the synodic and anomalistic months. In 2024/2015 during Minor Lunar Standstill and Saros #137, such oscillations of exceptional strength have been observed (*56*). Furthermore, the Moon’s pull on the Earth‘s crust leads to variations in the ground electric field, radon emanation, and microseismic vibrations (*57*). Radon can induce strong transient Ca^2+^ fluxes by bystander effects in the brain including the mammalian circadian clock in the hypothalamus (*58*), which might also control circalunar rhythms. Future investigations have to show whether there is a causal relation between geophysical rhythms and the menstrual cycle.

### Comparison with other species and putative impact on reproductive physiology

The number of reports demonstrating a coupling of reproductive cycles to the synodic month in terrestrial animals is steadily increasing although it is not always clear why reproductive cycles should be related to the Moon. Wild Eurasian badgers (*Meles meles*) mate around new Moon (*9*), which appears to be of selective advantage, because, at this time, it is least likely to be seen by predators. Similarly, conception rates of tropic cattle from farms in Venezuela were highest shortly before new Moon, while the frequency of calving was highest at new and full Moon (*11*). In domestic Holstein cattle monitored in farms in Hokkaido (Japan), calving frequency showed only one peak that occurred briefly before full Moon (*10*). One may argue that during full Moon predators can be seen much earlier that during dark nights. This is especially important for larger animals such as cows that have more difficulties to hide than small badgers. African lions hunt mainly during the first half of the night and, especially, in the weeks following full Moon (the waning Moon), when the Moon raises after sunset and it is dark at the beginning of the night (*59*). This might be true also for other predators. In Tanzania, most humans were killed by lions during the waning Moon when the beginning of the night is dark (*59*). In humans, the time between ovulation and menses lasts on average 12.4 days (*60*), meaning that those individuals with menses onset 1 to 2 days before full Moon had their most fertile phase before the new Moon when it is dark and dangerous to be outside in natural environments (*21*). There might have been a selection for humans who avoided predation by staying in a secure refuge around the time of the new Moon, and those humans might have used this time for reproduction. Macaques, gorillas, and orangutans also have menstrual cycle lengths of about 30 days (*61*, *62*). In macaques, the fertile phases appear coupled to new Moon (*13*), while, in mountain gorillas that have no natural enemies, it seems rather to couple to full Moon (*21*, *63*). It will be most promising to investigate these relationships in other terrestrial animals and in more detail in the future.

In summary, we show here that women can entrain to lunar cycles and that the synodic month with its prominent nocturnal luminescence cycles acts as the strongest zeitgeber. We also show that light pollution that has increased markedly during the past decades together with our changed lifestyle, which leads to an increased exposure to artificial light at night has strongly weakened the entrainment of the menstrual cycle to the lunar cycle. Artificial light at night not only obscures natural moonlight cycles but also shortens the length of the menstrual cycle (*21*, *33*–*35*). This shortening, in turn, reduces the probability of synchronization with the lunar cycle, as ongoing synchronization is only possible if the length of the menstrual cycle is close to that of the lunar cycle. Because menstrual cycle length appears to be an age-dependent marker of female fertility (*64*), our findings may prove to be relevant not only to human physiology and behavior but also to fertility and contraception (*21*).

## MATERIALS AND METHODS

### Subjects and data processing

All data (176 sets) belonged to women living in the Northern Hemisphere (latitudes ranging between 31°N and 51°N) who had recorded the onset of their menses out of personal interest (except for those recorded in Italy before 2000, vide infra), starting at an age ranging from 13 and 36 years, and were not on contraceptive medication. These sets were obtained:

1) From women who provided them by spontaneously contacting author C.H.-F. after they had learnt of their interest in diaries of menstrual cycles. In some instances, these data were provided as copies of calendars, in which the onset of menses was noted as a cross, in others as a list of menses onsets recorded on paper (example in Fig. 1) or in electronic format. In some cases, the records included information on births and/or miscarriages.

2) From registered public health initiatives/information campaigns on fertility themes.

3) The data for the Italian women collected before 2000 are part of the “Billings” subset of the Bernardo Colombo database (www.stat.unipd.it/ricerca/basi-di-dati) (*65*), which was interrogated having acquired permission from the Department of Statistics of the University of Padova through standard procedure. Only data from women who had recorded their menses continuously for a period of more than two years were used.

Institutional review board approval was sought for (Würzburg University, 20210407 01) to store, collate, and retrospectively analyze all available anonymized data. Written informed consent had been obtained in all instances, for each relevant dataset/database.

### Generation of mensograms

Moon phases (full and new Moons), apogees and perigees, and minimal and maximal lunar standstills were downloaded for each year between 1950 and 2024 from http://astropixels.com/ephemeris/moon/moondec1901.html or http://astropixels.com/ephemeris/moon/moondec2001.html with the courtesy of Fred Espenak and copy pasted to Notepad++. Only the relevant information such as date (year, month, and day) and time (in full hours) was transformed into a CSV file. The CSV file was then imported into a common Microsoft SQL-Server database. A spreadsheet was generated, in which the first column contained date/time (from 1 January 1950, 00:00 until 31 December 2024, 23:00) in 1-hour steps in consecutive lines. The next six columns contained the dates/times of lunar events (perigee, apogee, full Moon, new Moon, and maximal and minimal lunar standstills). The time points of lunar events were approximated to full hours and set to “1” at the relevant date/time. All other values in the columns were set to “0.” The spreadsheet with the lunar data was opened in Excel and the onsets of menses for all women were entered manually (as 1) in consecutive columns, respectively. Because we had no information about the exact times of menses onset, we entered 1 for each hour of the respective day. The file was then exported as txt-file and uploaded in ActogramJ (*30*) to display the data of each woman as mensogram. The mensograms were plotted with periods close to the synodic and tropical months together with the lunar data. This facilitated the visual detection of any correlations of lunar events with menses onsets.

### Determination of menses period and limits of entrainment

All mensograms were visually inspected, and phase jumps were determined by eye. For recorded intervals at which no phase jumps occurred and the menstrual period appeared stable by visual inspection, we calculated the period length with Lombscarcle-periodogram analysis software embedded in ActogramJ (*30*). To calculate the limits of entrainment, the period lengths before or after a phase of synchronization with one of the three lunar cycles were taken as the period at which the cycle could entrain (Fig. 4). In individual women several such entrainment phases could occur that were all included in the calculation. An entrainment phase was defined as a coupling of menses onsets to the lunar cycles for at least 4 consecutive months. As can be observed in individual mensograms, menstruation occurred quite frequently for 2 consecutive months at full Moon/new Moon, apogee/perigee, or minimum/maximum lunar standstill, sometimes after sudden phase jumps. The fact that it occurred in the same phase in 3 consecutive months already suggested entrainment. However, to be more conservative and reliably exclude random events, a threshold of 4 (rather than 3) months was chosen to define synchronization/entrainment. For each woman, we then calculated a mean of all periods occurring within the entire recording time. These mean values served for the calculation of means for all women (Table 1).

### Circular plots and Rayleigh test

For determining the phase distribution of the menses onsets in relation to the lunar phases, we plotted the data in circular plots and tested for deviation from a uniform distribution with the Rayleigh test using the statistics software R (*66*) as described previously (*21*). In brief, we converted the dates of menses onsets to cyclic data within the different Moon cycles by creating hourly menstrual dates, which were normalized to the exact length of the time interval between the previous and next full Moon (or perigee or maximal lunar standstill, respectively); resulting values were then converted to radians by multiplying them by 2π. Because the standard Rayleigh test can discriminate between a uniform (null hypothesis) and a unimodal frequency distribution but menstrual cycles could be locked either on the full or the new Moon and thus be axial bimodal, we applied phase duplication to the data so that both data close to the full Moon or close to the new Moon will fall into the same residual class (thus transforming an axial bimodal into a unimodal distribution) (*67*). Two separate Rayleigh tests, (i) for any deviations from a uniform frequency distribution of any phase and (ii) for the particular presence of a uni- or bimodal distribution with the phases synchronized to a central parameter 0 (e.g., the full/new Moon, perigee/apogee, or maximum/minimum lunar standstill, respectively), were then carried out for each woman using the R package “circular” from C. Agostinelli and U. Lund: Circular Statistics (version 0.4-93) (available at https://cran.r-project.org/web/packages/circular/citation.html) as well as pooled data (see below). In the circular plots, we provide *P* values estimated from the Rayleigh tests as well as the test statistics *S*, i.e., the length of the directional vector; larger values of *S* indicate a stronger concentration of menses on a particular Moon phase.

For the graphical presentation, we split the range 0 to 2π into 30 equally sized segments (one segment ~1 day for the synodic and ~22 hours for the anomalistic and tropical cycle). We created separate circular plots for each woman in relation to the relevant phases of the lunar cycles (full/new Moon, apogee/perigee, and maximum/minimum lunar standstills) (Fig. 1C). We also pooled the data from all women (separately for the synodic, anomalistic, and tropical months) and separately visualized and analyzed data falling into the two winter months: December and January (Fig. 6). Furthermore, we analyzed the pooled data separately according to whether they were collected before or after 2010 (Fig. 7). Last, we compare the pooled data over the whole period with menses data collected just in the 3 years around either Minor or Major Lunar Standstills (Fig. 8).
