# Supplementary material for: Synchronization of women’s menstruation with the Moon has decreased but remains detectable when gravitational pull is strong
Source: Sci Adv. 2025 Sep 24;11(39):eadw4096. doi: 10.1126/sciadv.adw4096 (PMC13141897; doi:10.1126/sciadv.adw4096)
Supplement: Supplementary file 1 — Figs. S1 to S6 [file sciadv.adw4096_sm.pdf]

Science Advances
AAAS

Supplementary Materials for

Synchronization of women's menstruation with the Moon has decreased but remains detectable when gravitational pull is strong

Charlotte Helfrich-Förster *et al.*

Corresponding author: Charlotte Helfrich-Förster, charlotte.foerster@uni-wuerzburg.de

Sci. Adv. **11**, eadw4096 (2025)
DOI: 10.1126/sciadv.adw4096

This PDF file includes:

Figs. S1 to S6

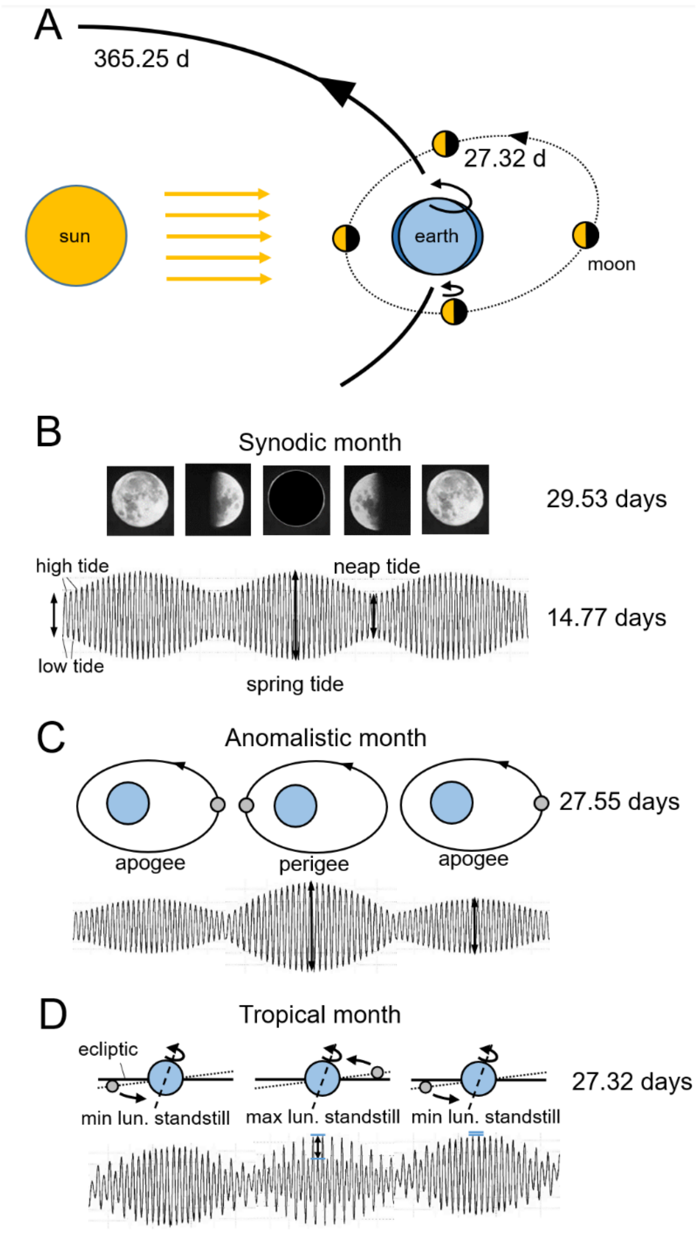

Fig. S1. The illuminance and gravimetric cycles that the moon imposes on earth. A. Schematic of the movements of moon and earth around the sun (sizes of sun, earth and moon and distances between them are not to scale). The earth takes 366,25 days to orbit the sun, while the moon needs 27.32 days to complete its elliptical orbit around the earth. **B**. The sun illuminates the moon, which causes a 29.53-day cycle of changing nocturnal illuminance on earth (full, half and new moon on earth depending on the position of the moon as depicted in A). The 29.53-day period between subsequent full moons is called synodic month. It is ~2.2 days longer than the orbital period of the moon, because the earth has moved on its orbit on the sun and it takes 2.2 days until the moon is again in a line with the earth and sun (its full moon position). In addition to nocturnal illumination, the moon exerts gravitational effects on earth that are most evident in the tides (dark blue shades around the earth in A). High and low tide occur twice a day with a period of 12.4 hours, but the amplitude of the tides depends on the position of the moon in relation to the sun and earth. The tides are maximal (spring tides), when earth, moon and sun are in one line (during moon-sun-earth syzygy). This happens during full and new moon, thus every half synodic month (14.765 days). During half-moon the tides are minimal (nip tides). **C**. The orbit of the moon around the earth is elliptic with the earth in one of the focal points of the ellipse. This means that the moon-earth distance cycles: every 27.55 days the moon is close to the earth (in its perigee) and exerts maximal gravitational forces on earth resulting in high tide amplitudes. Every 27.55 days the moon is far from the earth (in its apogee) and exerts minimal gravitational forces on earth resulting in low tide amplitudes. This cycle is called anomalistic month. The reason for the difference between moon's orbital period and perigee-to-perigee period is a cycling of the moon's elliptical orbit around the earth every 9.25 years. **D**. The moon's orbit around the earth is tilted in respect to the plane of the earth's orbit around the sun (the ecliptic). When the moon is high in the northern sky (in its tropic), the difference in the amplitude between the two daily tides is maximal in the northern hemisphere of the earth. This happens every 27.32 days (moon's orbital period) and the corresponding cycle is called tropical month.

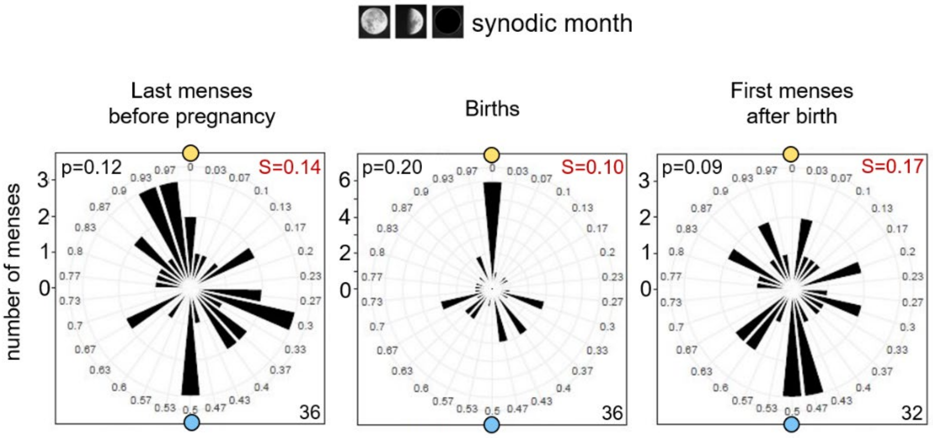

Fig. S2. Distribution of the last menses before pregnancy, the births and the first menses after birth. All events were quite peaked (all S values ≥10) occurring before full or new moon (last menses before pregnancy), around full moon (births) and around new moon (first menses after birth). However, due to the small number of cases this did not reach significance (n=36 for the menses before pregnancy and births, respectively, and n=32 for the menses after birth as 4 women stopped to record their menses after their dream of having children came true).

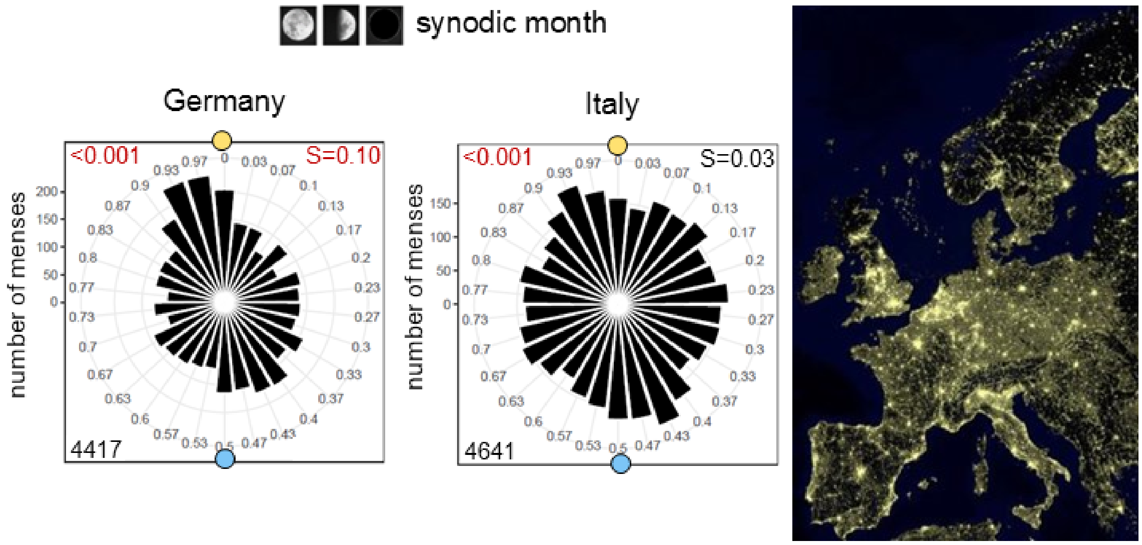

Fig. S3. Polar phase plots of the menses of the German and Italian women. Circular phase plots show the phase relation between menstrual cycles and the synodic month. These phase plots contain all menses from the German or Italian women, respectively (before and after 2010). The plots show that the phase distribution is less peaked for the Italian women. **D.** Satellite picture of Europe at night from "Earthlights 2002" published by NASA.

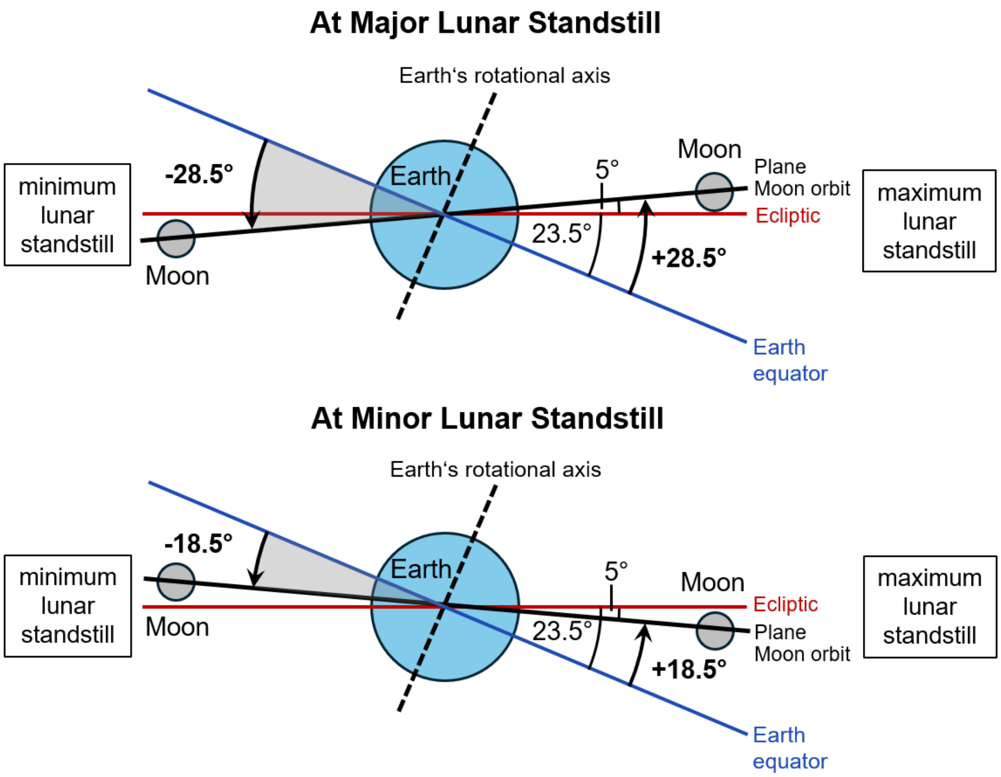

Fig. S4. Major and Minor Lunar Standstills. The Moon needs 27.3 d for one revolution around the Earth (= the tropical month). Since its orbit is tilted in respect to the Earth's orbit around the sun (the ecliptic) by 5.14°, the Moon reaches low and high altitudes in the sky every half tropical month (minimum and maximum lunar standstills). Thus, the moon's declination varies cyclically with a period of 27.3d. In addition, the spatial direction of the Moon's orbital inclination gradually changes over an 18.6-year cycle, leading to a change in the amplitude of the 27.3d oscillation. Every 9.3 years, the amplitude reaches its maximum (= Major Lunar Standstill) and its minimum (= Minor Lunar Standstill), respectively. At Major Lunar Standstill the declination of the Moon in respect of the Earth's equator is ±28.6° (5.14° more than the earth's axial tilt) while it is ±18.3° (5.14° less than the earth's axial tilt) at Minor Lunar Standstill. Moon and Earth are shown from the side and the distance between Moon and Earth is not to scale. Redrawn after Wikimedia Commons (https//upload.wikimedia.org/wikipedia/commons/b/b6/Lunar_standstill.GIF).

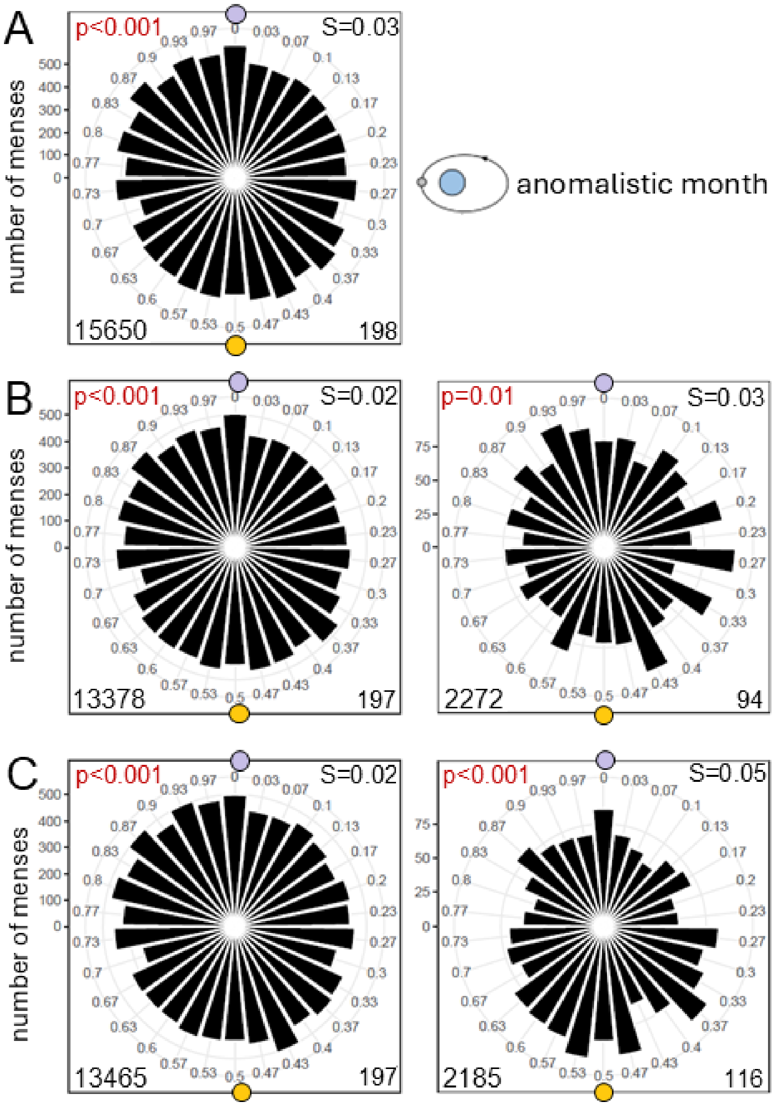

Fig. S5. The alignment of menstrual cycles with the anomalistic month is only slightly influenced by Major and Minor Lunar Standstills. A. Polar phase plots of all menses onsets (including the old and new study) relative to anomalistic months for the entire recorded period (1950-2024). **B.** Menses distribution outside (left) and within 3-year intervals around Minor Lunar Standstill (1979, 1997, 2015) (right). **C.** Menses distribution outside (left) and within 3-year intervals around Major Lunar Standstill (1987, 2006, 2025). For the major standstill that will occur in March 2025, we used the years 2023-24. Menses distribution was slightly more peaked and directed toward apogee during Major Lunar Standstill than at other times. Labeling as in Fig. 8 of the main manuscript.

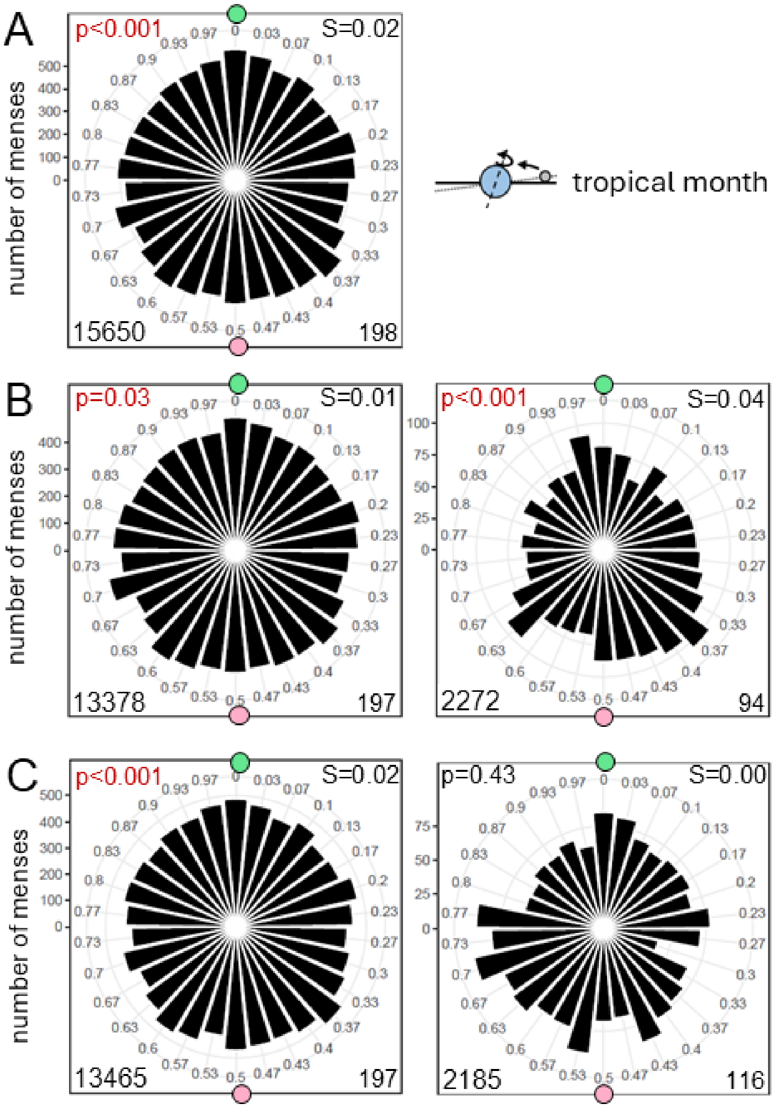

Fig. S6. The alignment of menstrual cycles with the tropical month is only slightly influenced by Major and Minor Lunar Standstills. A. Polar phase plots of all menses onsets (including the old and new study) relative to tropical months for the entire recorded period (1950-2024). **B.** Menses distribution outside (left) and within 3-year intervals around Minor Lunar Standstill (1979, 1997, 2015) (right). **C.** Menses distribution outside (left) and within 3-year intervals around Major Lunar Standstill (1987, 2006, 2025). For the major standstill that will occur in March 2025, we used the years 2023-24. Menses distribution was slightly more peaked and directed toward minimum lunar standstill during Major Lunar Standstill than at other times. Labeling as in Fig. 8 of the main manuscript.
